# What keeps historical theme park visitors coming? Research based on expectation confirmation theory

**DOI:** 10.3389/fpsyg.2024.1293638

**Published:** 2024-02-29

**Authors:** Li Yuan, Azizan Marzuki

**Affiliations:** School of Housing, Building and Planning, Universiti Sains Malaysia, Penang, Malaysia

**Keywords:** historical theme parks, expectation confirmation theory, tourism experience, satisfaction, structural equation model

## Abstract

Culture is one of the most important factors in attracting tourists and influencing the tourist experience. In China, “theme park” is a new field of tourism research, an excellent theme park can not only drive the development of a city’s tourism industry, but also help it better publicize its history and culture. The article takes Kaifeng, China as the background, and selects the Song Dynasty historical and cultural theme park, which was established based on the traditional ink painting Qingming Riverside Drawing, as an example, to study the factors affecting tourists’ travel experience in historical and cultural theme parks, and based on which it explores the reasons for the formation of tourists’ satisfaction and post-trip behavioral intentions, to provide references and suggestions for the development of cultural theme parks from the point of view of travel experience. Based on the expectation confirmation theory, this essay introduces five constructs, namely, expectation (tourism motivation), performance (service quality), confirmation (tourism experience), satisfaction, and post-trip behavior intention, to construct a model of the factors influencing tourists’ satisfaction by tourism experience in historical theme parks and analyze the intrinsic correlation among the factors within the model. It is found that tourists’ tourism experience is mainly affected by tourism motivation and service quality, satisfaction and post-trip behavior intention are jointly determined by the above three. According to the research results, this study believes that tourism motivation, service quality, and tourism experience should be the focus of attention of the current tourist destinations, therefore, focusing on tourists’ motivation to optimize the quality of service, helping tourists to enhance the sense of tourism experience, and improving based on the above focuses is of great significance to promote the development of historical and cultural theme parks.

## 1 Introduction

Tourism is an industry of continuous innovation and development, which changes constantly with the needs of tourists. Most tourist destinations attract tourists through rich tourism resources and good service quality, and provide a good tourism experience, in order to ensure tourist satisfaction and loyalty, to stand out from a large number of tourist destinations, so more and more scholars in the tourism industry are committed to studying the reasons why people are willing to revisit and recommend the destination (Liu et al., 2016).

In recent years, travel has become progressively more popular, and travelers have begun to seek more unique travel experiences (Poon, 1994). People’s motivations for traveling have also increased, from an initial desire to escape from everyday life to a desire to enjoy experiences and experience culture while traveling (Krippendorf, 1986). Tourists’ sense of travel experience significantly affects their evaluation, satisfaction and post-trip behavior intention (Kim, 2010); Therefore, only by providing tourists with a travel experience that makes them feel novel, relaxed and pleasant can tourism enterprises’ income be guaranteed. In addition, theme parks are an important destination for leisure and entertainment in the tourism industry (Pikkemaat and Schuckert, 2007), which focus on providing tourists with a sense of fantasy and escapism, and the rise of theme parks has also enriched the way tourists experience tourism (Zhu et al., 2022). At the same time, some studies show that people today want to participate in tourism activities more than single-visit tourism in the past and are more willing to pay for unique and memorable tourism experiences (Duan et al., 2018), so the willingness of tourists to choose theme parks as tourism destinations is also increasing (Maccannell, 1976). The study of theme park tourism is of great significance and value for both tourists and tourism enterprises, both at present and in the future.

In summary, this paper expects to understand tourists’ experiential feelings by studying historical and cultural theme park tourism, focusing on tourists’ post-tour behavioral intentions, and exploring the degree of consumer satisfaction with historical and cultural theme park tourism by analyzing factors such as motivation and service quality. This research angle is relatively novel in the current related field, which helps tourism enterprises to understand the motivation behind tourists’ tourism behavior, and through the comparison between the degree of tourists’ expectation of the motivation and the perceived real service quality, to gain an in-depth understanding of the tourists’ feelings about the tour experience, and to explore the factors affecting the satisfaction and post-tour behavioral intentions. In today’s China, where theme park tourism is on the rise and competitiveness is increasing (Chen F. et al., 2022), the quality of tourism in theme parks is an important aspect that helps them to improve their competitiveness and ensures that tourists continue to play, and is therefore of particular importance (Akel and Çakir, 2022).

Therefore, this study is expected to provide the government, tourism enterprises, or investors with an in-depth analysis of the development of historical and cultural theme parks from the tourists’ perspective, and to provide suggestions and decision-making directions of great value. Through this study, we expect to understand the development prospects of historical and cultural theme parks, and deeply explore the critical factors of tourist demand, service quality improvement and development prospects, to provide more profound insights and contributions to the future development of Chinese theme parks (Rafei et al., 2022).

## 2 Theoretical framework, research hypotheses, and model construction

### 2.1 Expectation confirmation theory (ECT)

Initially, ECT was proposed by Oliver in the literature of the marketing field, which is mainly used to understand customer satisfaction and repeat purchase willingness. It explains the satisfaction generated by human expectations, perceived performance and the degree of expectation confirmation after a purchase (Zhao and Lu, 2018). Currently, ECT is widely used in tourism, service and experience research, such as tourism hotel experience (Boo and Busser, 2018), and urban tourism (Basil Chibuike et al., 2021). In particular, this theory is used in the study of tourism hotel experience to explore the relationship between satisfaction and intention to stay and revisit reached after a series of experiences with the hotel’s products and services for consumers (Boo and Busser, 2018). There are also numerous tourism countries that study the reasons for the emergence of tourists’ post-trip behavioral intentions based on ECT (Basil Chibuike et al., 2021).

In summary, the application of Expectancy Confirmation Theory (ECT) in tourism-related research fields is feasible and relatively mature; therefore, it is reasonable to use ECT as a theoretical framework to explore the factors influencing the tourism experience, tourist satisfaction and post-trip behavioral intentions in historical and cultural theme parks in this study.

### 2.2 Perceptual attributes of expectancy confirmation theory

#### 2.2.1 Expectations (tourism motivation) (TM)

Travel motivation is a kind of expectation that travelers have about their future travel experience (Boluk et al., 2017). For example, before traveling, people who are psychologically motivated to escape from their daily lives will pay more attention to other people’s comments about the immersiveness or degree of detachment from reality of the destination’s atmosphere, and try to obtain more relevant information through advertisements, etc., try to choose a location that will satisfy their needs (Pearce and Lee, 2005). The combination of the strength of psychological motivation and pre-trip information will produce a certain degree of attraction for tourists, and tourists will also produce pre-trip perceptions of the image of the destination, such as perceptions of the degree of immersion in the destination environment, or perceptions of the degree of excitement of amusement rides, which is the travel expectations of tourists generated by the psychological and external factors that are influenced by the factors that motivate tourists to carry out tourism activities, i.e., the tourism motivation., i.e., tourism motivation (Seabra et al., 2016; Zhao and Lu, 2018), therefore, expectations in this study will be regarded as tourism motivation. Tourism motivation serves as the beginning of tourism, which reflects the traveler’s initial thoughts and travel aspirations before starting a period of tourism activities (Caber and Albayrak, 2016), and is the internal and external motivation for people to generate tourism behaviors (Baloglu and Uysal, 1996).

Zhao Haisol believes that the first aspect of tourism motivation is the scenic attraction (SA), such as advertisements displaying the scenic environment and program content, which can only enhance the interest of tourists if they have a specific understanding of the scenic area (Zhao and Lu, 2018). Disneyland is good at publicizing and displaying the scenes and projects in the park through animated advertisements and other means, and has thus gained high popularity and awareness (Fung and Lee, 2009). Secondly, Kruger and Pearce suggest that novelty (NV) is also one of the critical tourism motivations for tourists (Pearce and Lee, 2005; Kruger and Saayman, 2010). People desire to satisfy their curiosity about unknown locations by exploring a new area. For example, thrill rides or shows in theme parks or parks can bring tourists different experiences and feelings (Johns and Gyimóthy, 2003). In addition, recreation and relaxation (RL) are one of the most important motivations for people to generate tourism behavior (Fodness, 1994; Pearce and Lee, 2005; Zhu et al., 2022), and in most cases, tourism is a temporary reversal of real life, which satisfies the psychological motivation of people who want to relax and unwind (Yang et al., 2023). Moreover, when tourists develop this motivation, they actively participate in tourism activities to seek pleasure, and tourist participation is significant in travel, affecting their satisfaction and perceived quality of experience (Seabra et al., 2016). On the other hand, Kruger and Richards suggested that exploring culture (CE) is an essential motivation for tourism (Kruger and Saayman, 2010; Richards, 2018), Cultural exploration aims to understand the destination’s history, customs, life forms and so on, which can be seen as the essence of tourism (Defining Cultural Tourism, 2016). Onome also found in his study that the motivation of tourists to travel to different destinations varies, that experiencing and appreciating the culture of a destination is an essential motivation for tourists who travel to tourist areas with certain cultural qualities, and that learning about different cultures is a way of self-actualization (Awaritefe, 2004). In addition, Qiao Guanghui believes that interpersonal relationship (IR) is an essential factor affecting the motivation to travel (Qiao, 2011). Studies have shown that the behavior of going on a trip with family and friends is a manifestation of wanting to socialize. Some interviewees said that they chose the long-distance train as a mode of tourist travel not only because the long-distance train is a means of transportation, but also because they want to get the opportunity to communicate with their family members and enjoy the time with their families (Hibbert et al., 2013), and that effective interactions between tourists and their families and friends in a tourist environment can positively influence tourists’ emotional experience and satisfaction (Chen H. et al., 2022).

In addition, tourism motivation is the psychological thoughts and demands of tourists on this trip before traveling, which contains certain activities and services expected by tourists (Thawornwiriyatrakul and Meeprom, 2020). For example, tourists who take leisure as their main travel purpose will pay more attention to whether the travel environment or atmosphere makes them feel comfortable and relaxed; tourists who take cultural experience as their main purpose will focus on whether the cultural atmosphere of the destination is strong or whether the culture they experience is unique. In summary, we believe that different types of motivation will cause tourists to focus on and experience service quality differently, which may have a clear impact on tourists’ perceptions of service quality while traveling. Second, if the provider continuously improves the service quality and tries to satisfy tourists’ pre-trip wants and needs, tourists get a better experience, which will enhance their satisfaction and lead to positive post-trip behavioral intentions (Lee et al., 2011).

Therefore, the prerequisite for effective tourism marketing is to understand the motivation of tourists (Fodness, 1994), and planners need to understand the reasons why tourists decide to engage in this consumption behavior in order to plan better and market the services and programs of the destination (Clancy and Lou Roberts, 1984).

#### 2.2.2 Perceived performance (service quality) (SQ)

Service quality is the overall experience of the services provided by the tourism industry to tourists on their trips (Obenour et al., 2006). Perceived performance in tourism mainly refers to the service quality inherent in the tourism area that tourists perceive during the tourism experience (Laihonen et al., 2014), which can be principally summarized as the quality of human services (Uzir et al., 2021), the conditions of the hardware and facilities, and the overall environmental atmosphere (Ryan et al., 2010), and the tourists’ perceptions of the objective services of the tourism area are the main focus of this paper. The feelings of tourists toward these objective services in the tourist area are the perceived performance in this paper. Therefore, the article is based on the research theme, and perceived performance is regarded as service quality. Secondly, in the past decades, numerous researchers have explored the factors affecting tourism experience and finally determined that service quality is the most critical aspect in determining tourists’ tourism experience, among which, Wang et al. proposed five critical factors in service quality: resource conditions (RC), recreational activities (RA), tourism facilities (TF), integrated management (IM) and related personnel (RP) (Palmer and O’Neill, 2003; Wang et al., 2012).

First, a destination’s resource conditions (RC) are essential factors that attract tourists and influence their experiences (Wang et al., 2012). For example, for travelers going to natural scenery areas, places with beautiful scenery and comfortable climate are their ideal destinations (Ross, 1991), and for tourists going to historical and cultural resorts, rich historical and cultural resources are an essential element of the tourist experience they want to have. Second is the entertainment activities (RA) in theme parks, which generally include performances, music, and other forms, which can assist the parks in displaying their themes better, and at the same time, have a strong interpretive role in publicizing and educating, learning history, and preserving heritage (Pearce, 2008). In addition, in the tourism environment, tourism facilities (TF) are a kind of “disturbing factor” that affects the quality of tourism services, which includes tourists’ concerns about inconvenient transportation, lack of infrastructure, poor accommodation, and so on, and the conditions of tangible resources can affect tourists’ experience and satisfaction to a greater extent than the more abstract tourism elements (Moscardo and Pearce, 1986). Therefore, providing adequate tourism facilities is crucial to eliminate the tangible “distractions” in the experience. Once again, the tourist attraction’s integrated management (IM) is essential. Failed management can lead to disorganization of the destination’s environment and people, destroying the tourist environment and uncivilized behavior of tourists, which can reduce the tourists’ sense of tourist experience. Therefore, maintaining its good image and regulating tourists’ destructive behaviors are vital factors (Su et al., 2016). Suppose the destination’s management can keep abreast of tourists’ expectations of scenic area management and make specific changes. In that case, it can alleviate tourists’ disappointment to a certain extent (Michalkó et al., 2015), to keep tourists’ destinations’ positive perceptions. Finally, there is the Relevant Person (RP) in the quality of service. During the trip, customers often complain because the service staff’s attitude is lower than expected, or some aspect of the experience is not pleasant. These incidents are often the result of improper management of the tourist area or the training of the staff is not qualified, so managers should actively understand the reasons for the tourists to complain, the staff should be trained adequately for the problem, and the park staff should be to exercise some control (Lin et al., 2017), in order to systematically optimize the tourism environment in tourists’ expectations (Park and Jeong, 2019).

High-quality service has always been the goal of the tourism industry, and the ability to provide satisfactory service affects the ultimate satisfaction and loyalty of consumers (Crompton and Mackay, 1989), so managers should ensure that the quality of service is high to maintain their competitive advantage.

#### 2.2.3 Degree of expectation confirmation (tourism experience) (CF)

Firstly, before the start of tourism activities, tourists will generate motivation (expectation) (Su et al., 2020). They will travel to the destination because they want to feel good service quality (perceived performance). Tourists may be attracted by other people’s evaluations of the destination or the destination’s promoted information, and based on this information, they will generate pre-trip perceptions of the destination, which will lead to the expectation of traveling to the destination (Wang et al., 2016). Secondly, only after tourists have identified the destination, traveled to the tour, and personally experienced the service quality, they can confirm the real service quality of the tour, finally, the real perceived performance and pre-tour expectations will be contrasted, which will lead to the formation of the sense of tourism experience. In ECT, the higher the expectations of tourists, if the actual performance does not exceed the expectations, the lower the degree of confirmation and indirectly affects consumer satisfaction, on the contrary, the original expectations are lower, and the actual performance is higher, the higher the degree of confirmation, and also indirectly increase satisfaction (Asmelash and Kumar, 2019). The degree of expectation confirmation is generated through the comparison of expectation and perceived performance, the article combines the theme that tourists need to compare the initial feeling of expectation (tourism motivation) and perceived performance (service quality) in order to contribute to the final sense of tourism experience, takes tourism experience as the degree of expectation confirmation in this paper, a better sense of experience represents a higher degree of expectation confirmation, and a poorer sense of experience represents expectation confirmation degree is lower. In addition, Wang et al. proposed in their study that the quality of experience takes behavioral, aesthetic and emotional experiences as the primary research factors in tourism experience (Wang et al., 2012), so the article will focus on the three dimensions of behavioral experience, aesthetic experience and emotional experience in tourism experience.

Among the dimensions of experiential consumption studied by Schmitt, including sensations, feelings, thoughts, behaviors and associations, behavioral experiences (CE) impact people’s experiences, lifestyles, and interactions, and can motivate their behaviors and inspire their lifestyles (Schmitt, 1999). Therefore, an excellent behavioral experience can enrich tourists’ emotions to a certain extent and positively impact tourists’ lives, thus positively influencing tourists’ satisfaction and post-trip behavioral intentions. Second is the aesthetic experience (AE); the aesthetic impression of the destination will have an impact on the overall tourism experience, and an excellent aesthetic environment can evoke positive emotions such as joy and surprise, which can help to increase tourist satisfaction and ensure that tourists are willing to revisit the destination again or recommend the destination to other people’s post-trip behavioral intentions (Kirillova and Lehto, 2015). In addition, Schmitt proposed the Strategic Experience Module to create different customer experiences, including emotional experience (EE), cognitive experience, physical experience, behavior and lifestyle (Schmitt, 1999). Tourists’ emotions play an important role in their cognitive evaluation of the tourism experience and their post-trip behavioral responses (Volo, 2021). It has been found that emotional experiences in imagination, daydreaming and expectations can have a significant impact on the tourist’s choice of destination (Goossens, 2000). It is this aspect of the experience that theme parks want to emphasize to tourists, which leads to the fact that tourists’ emotions assume an essential role in the creation of the tourist experience (Niu et al., n.d.), and in comparison with other tourist experiences, historical culture and nostalgia are stronger in cultural theme parks than in other tourist experiences. It enables tourists to develop personal emotions related to the theme (Smith and Conrad, 2020).

In contemporary theme parks, the theme that runs through the whole is mainly expressed through the park’s landscape, architecture, facilities, performances, special costumed personnel, and specialty food and beverage, each affecting the visitor experience (Milman et al., 2012). Therefore, theme parks should continuously innovate to bring new experiences to visitors’ senses, such as new rides, facilities, and performances, and so on. When visitors’ positive emotions are stimulated in an objective environment, their satisfaction with the destination will increase (Ma et al., 2013).

#### 2.2.4 Satisfaction (SF)

Consumer satisfaction depends on the overall attitude resulting from purchasing and experiencing a product (Tandon et al., 2020). In traveling, it is the reaction of tourists after they have had a travel experience (Asmelash and Kumar, 2019).

On the other hand, satisfaction is an important factor that influences tourists to revisit and recommend tourist destinations, and studies have shown that the higher the satisfaction level, the stronger the willingness to revisit and recommend (Rajesh, 2013). For tourist destinations, making tourists feel happier and more satisfied than unpleasant feelings during the tour can lead to tourist satisfaction (Hui et al., 2007). However, unlike a simple consumer product, tourism consists of many different segments, including accommodation, food, and recreational activities. Because these segments are interrelated, there may be a situation where good or bad performance in one segment leads to overall satisfaction or dissatisfaction.

Therefore, when judging satisfaction, tourism managers must carefully analyze what factors can help to increase tourist satisfaction, and the impact caused by each component should be measured and recognized (Pizam et al., 1978).

#### 2.2.5 Post-trip behavioral intention (BI)

In general, positive post-trip behavioral intentions include the willingness to revisit and promote the destination to others after a complete series of travel experiences, and tourists’ loyalty to a destination is often reflected in their willingness to revisit and recommend it as well (Chen and Tsai, 2007; Ali et al., 2016). At the same time, studies have shown that the higher the satisfaction level, the higher the likelihood of consumer repurchase and spontaneous word-of-mouth publicity, and some surveys have found that many tourists choose their destinations based on the recommendations and introductions of family members or friends (Chen and Chen, 2010). Therefore, understanding tourists’ post-trip behavioral intentions can better help managers learn how to build a more attractive tourism image and improve the problems that arise to maximize tourism resources (Chen and Tsai, 2007; Jahanger et al., 2022).

### 2.3 Research hypothesis

Based on the Expectation Confirmation Theory model, this study explores the influential relationship between expectation (tourism motivation), perceived performance (service quality), degree of expectation confirmation (tourism experience), satisfaction, and post-trip behavioral intention.

Based on the above discussion, the article makes the following hypotheses:

*H1:* Tourists’ tourism motivation positively affects their perception of tourism service quality.

*H2:* Tourists’ perception of tourism service quality positively affects their tourism experience.

*H3:* Tourists’ expectation of tourism motivation positively affects their tourism experience.

*H4:* Tourists’ tourism experience positively affects their satisfaction.

*H5:* Tourists’ satisfaction with tourism positively affects their post-tour behavioral intention.

### 2.4 Model construction

**Figure 1:**
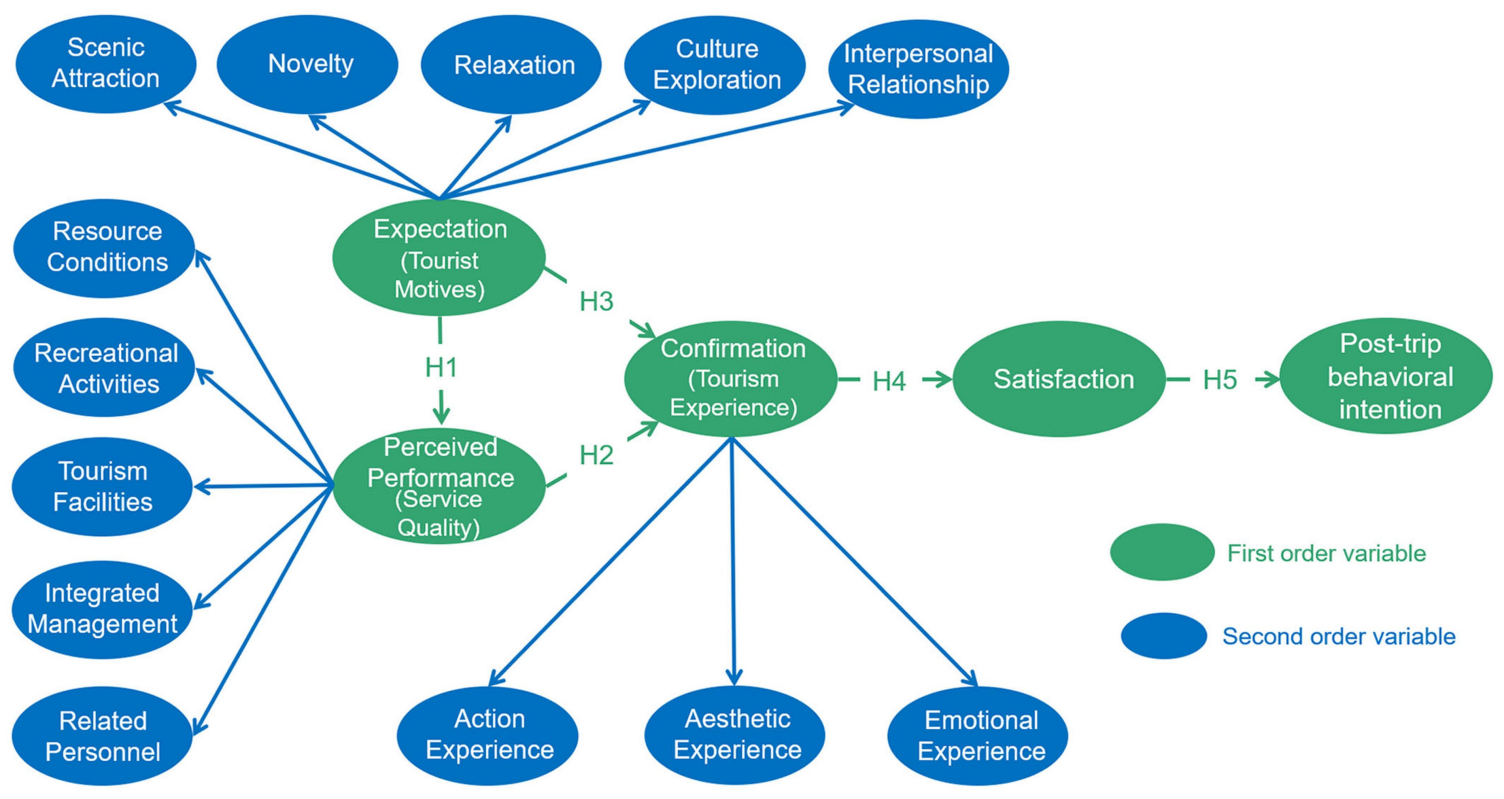
Research structure.

Based on the above assumptions and related discussions, the theoretical model framework can be drawn as Figure 1.

## 3 Research design and methodology

### 3.1 Questionnaire design

In order to ensure the accuracy and reliability of this study, the scales referred to in the article have all been verified by relevant literature, and on this basis, the corresponding contents have been adjusted and optimized to a certain extent according to the research topic, and the questionnaire questions were finally designed. The questionnaire is divided into two parts: the first part is to collect the basic information of the respondents, and the second part is to measure the tourism behavior of the tourists in Qingming Shangheyuan. The latent variables within the questionnaire were all based on a seven-point Likert equidistant scale, where “1” is completely disagree, “2” is disagree, “3” is relatively disagree, “4” is average, “5” is relatively agree, “6” is agree, and “7” is completely agree. Respondents made their choices based solely on their actual travel feelings. The operational definitions, question items, and scale references for this study are shown in Table 1.

**Table 1:** Definition of variable operability and reference scales.

Research variables (First)	Operational definitions	Literature sources
Expectation (Tourist Motives)	The motivation that drives tourists to travel to Qingming Shangheyuan before the trip is also the expectations that tourists create for the overall trip.	Zhao and Lu (2018)
Performance (Service Quality)	It refers to the performance of visitors’ perception of the quality of various services in Qingming Shangheyuan.	Wang et al. (2012)
Confirmation (Tourism Experience)	It is the experience and feeling of the tourists when they make a trip in Qingming Shangheyuan.	Wang et al. (2012)
Satisfaction	Refers to the level of satisfaction of visitors with their overall trip to Qingming Shangheyuan.	Ali et al. (2018) and Zeng and Li (2021)
Post-trip behavioral intention	Refers to the likelihood that a tourist will revisit or recommend a destination in the future after completing a trip.	Wang et al. (2012), Ali et al. (2018), Cheng et al. (2015), Zeng and Li (2021), and Zhang and Walsh (2020)
Scenic Attraction	Refers to what attracts visitors to the Qingming Shangheyuan itself.	Awaritefe (2004) and Zhao and Lu (2018)
Novelty	Refers to the sense of novelty and curiosity that tourists feel about Qingming Shangheyuan before traveling.	Kruger and Saayman (2010), Zhang and Walsh (2020), and Kim (2010)
Relaxation	Refers to the expected psychology of a tourist who wants to engage in leisure and relaxation before traveling.	Kay (2009) and Yang et al. (2023)
Culture Exploration	Refers to the psychology of expectations of tourists who want to learn and explore the culture before traveling.	Kay (2009) and Zhao and Lu (2018)
Interpersonal relationship	Refers to the psychology of the tourist’s expectations about relationships on this trip before the trip.	Zhang and Walsh (2020) and Qiao (2011)
Resource conditions	Refers to the extent to which visitors perceive the adequacy of resource conditions in the Qingming Shangheyuan.	Zeng and Li (2021), Wang et al. (2012), and Zhang and Walsh (2020)
Recreational activities	Refers to the overall feeling of visitors toward the entertainment activities held in Qingming Shangheyuan.	Wang et al. (2012) and Zhang and Walsh (2020)
Tourism facilities	Refers to the degree to which visitors feel that the facilities in the park are effective and adequate.	Wang et al. (2012)
Integrated management	Refers to the extent to which visitors feel that the controls in Qingming Shangheyuan are satisfactory.	Wang et al. (2012) and Cheng et al. (2015)
Related personnel	Refers to the visitor’s overall perception of the behavior and demeanor of the people in the park.	Wang et al. (2012), Ali et al. (2018), and Kim (2010)
Action experience	Refers to the fact that the tourists have thought about their lives or behavior in some way as a result of the tour.	Wang et al. (2012), Milman and Tasci (2018), and Zhang and Walsh (2020)
Aesthetic experience	Refers to the visitors’ aesthetic perception of the overall environment of Qingming Shangheyuan.	Wang et al. (2012), Ali et al. (2018), and Zhang and Walsh (2020)
Emotional experience	Refers to the emotional experience that visitors get during their trip to Qingming Shangheyuan.	Zeng and Li (2021)

### 3.2 Object of study

The theme of this study is to explore the factors affecting tourism experience, satisfaction and post-trip behavioral intention in theme parks and to use Qingming Shangheyuan as a research case. The reason is that the primary purpose of Chinese theme parks is to spread Chinese culture, which is different from most Western theme parks that intend to help tourists to get away from reality and worries, so the Chinese cultural elements play a significant role in the construction of these parks (Yang, 2011). For example, the first theme park in Shenzhen, China, “Splendid China,” has been described as “a window on China’s history, culture and heritage” (Sofield and Li, 1998, p. 382), and has attracted up to 3 million tourists annually since it first opened in 1989 (Zhang et al., 2021). The attraction of historical and cultural themes for tourists is obvious. Since then, other theme parks gradually began to emerge in China, and with economic globalization, people’s thinking and consumption patterns have been influenced by the West. Chinese theme parks are no longer limited to cultural and ideological education and the promotion of national identity, but have begun to increase the function of recreation and entertainment. This change has made China’s historical and cultural theme parks more readily accepted and enjoyed by the public (Zhang and Shan, 2016). Qingming Shangheyuan, on the other hand, integrates the traditional culture of China’s Northern Song Dynasty with modern leisure and recreational facilities, which enhances the modernity and interest of historical and cultural-themed trips based on spreading Chinese culture. Therefore, the article selects Qingming Shangheyuan as a study case.

On the other hand, due to the broad coverage of tourists in the tourism environment, the gender, age, and occupation of the survey respondents are not restricted in this survey, in order to better collect the real feelings and thoughts of different groups of people in the theme park tourism, so that the survey results can be more comprehensive. In order to ensure the authenticity and validity of the questionnaires, the survey respondents are all the tourists who have been to travel to the Qingming Shangheyuan. Secondly, all fillers scanned or clicked on the QR code or URL link of the questionnaire to view the survey instructions of the study. The survey participants volunteered to answer the research questions and could withdraw from question completion anytime. Therefore, all participants agreed, were fully informed, and participated voluntarily in completing this questionnaire.

### 3.3 Data collection

The questionnaire of this study was distributed in Kaifeng City. In this city, Qingming Shangheyuan is located, and at the same time, it was collected in combination with several online platforms. Bentler and Chou in 1987 proposed a sample size should be at least 5 times that of the estimated parameter (under normal conditions, without missing or exceptional values) (Bentler and Chou, 1987). The survey time of the questionnaire of this study is from May to July 2023, and a total of 713 samples were received, and the sample size after excluding invalid questionnaires is 425. It is 8 times more than the estimated parameter (52), applicable to this study. The basic information of the respondents is shown in Table 2.

**Table 2:** Descriptive statistics.

Sample	Category	Number	Percentage
Gender	Male	189	44.5%
	Female	236	55.5%
Age	Under 20	40	9.4%
	21–30	183	43.1%
	31–40	84	19.8%
	41–50	66	15.5%
	Over 51	52	12.2%
Monthly income (RMB)	Under 4,000	162	38.1%
	4,001–8,000	126	29.6%
	8,001–12,000	74	17.4%
	12,001–16,000	37	8.7%
	16,001–20,000	10	2.4%
	Over 20,000	16	3.8%
Education	Junior high school or below	20	4.7%
	High school or junior college	41	9.6%
	Bachelor’s degree or college	285	67.1%
	Master’s degree	69	16.2%
	Doctor or above	10	2.4%
Occupation	Manufacturing	26	6.1%
	Medical	25	5.9%
	Finance	39	9.2%
	Design	32	7.5%
	Service Industry	59	13.9%
	Education	112	26.4%
	Students	67	15.8%
	Freelance	16	3.8%
	Others	49	11.5%
Marital status	Unmarried	193	45.4%
	Married	232	54.6%
Number of entry	1 time	240	56.5%
	2 times	88	20.7%
	3 times and above	97	22.8%
Location	Inside Henan Province	162	38.1%
	Outside Henan Province	263	61.9%

### 3.4 Results of reliability analysis

In this study, SPSS 24.0 was used for reliability analysis to measure the degree of consistency or stability of the results; as shown in Table 3, the Cronbach’s α coefficients of each measurement variable ranged from 0.739–0.904, which were all greater than 0.6, and the Cronbach’s α if Item Deleted values are not higher than the current Cronbach’s α value results, and Corrected Item-to-Total Correlation values are all greater than 0.5, which indicates that the scale in this study has good reliability.

**Table 3:** Results of confidence analysis.

Dimension	Items	Corrected Item-to-Total Correlation	Cronbach’s α if Item Deleted	Cronbach’s α
SA	SA1	0.662	0.624	0.770
	SA2	0.585	0.717	
	SA3	0.584	0.726	
NV	ID1	0.550	0.726	0.761
	ID2	0.639	0.626	
	ID3	0.592	0.682	
RL	RL1	0.674	0.844	0.865
	RL2	0.737	0.819	
	RL3	0.747	0.814	
	RL4	0.700	0.834	
CL	CL1	0.668	0.796	0.838
	CL2	0.709	0.778	
	CL3	0.634	0.813	
	CL4	0.673	0.794	
IR	IR1	0.617	0.611	0.739
	IR2	0.588	0.640	
	IR3	0.540	0.732	
RC	RC1	0.638	0.739	0.802
	RC2	0.600	0.782	
	RC3	0.709	0.665	
RA	RA1	0.679	0.765	0.827
	RA2	0.684	0.762	
	RA3	0.693	0.754	
TF	TF1	0.721	0.851	0.878
	TF2	0.751	0.839	
	TF3	0.753	0.837	
	TF4	0.725	0.848	
IM	IM1	0.670	0.718	0.805
	IM2	0.710	0.672	
	IM3	0.587	0.798	
RP	RP1	0.652	0.811	0.843
	RP2	0.671	0.805	
	RP3	0.721	0.781	
	RP4	0.671	0.804	
CE	BE1	0.759	0.883	0.904
	BE2	0.759	0.883	
	BE3	0.742	0.886	
	BE4	0.742	0.886	
	BE5	0.795	0.875	
AE	AE1	0.777	0.851	0.890
	AE2	0.748	0.862	
	AE3	0.754	0.860	
	AE4	0.752	0.861	
EE	EE1	0.656	0.751	0.813
	EE2	0.685	0.720	
	EE3	0.649	0.757	
SF	SF1	0.699	0.776	0.838
	SF2	0.707	0.768	
	SF3	0.695	0.780	
BI	BI1	0.625	0.816	0.826
	BI2	0.727	0.716	
	BI3	0.700	0.744	

### 3.5 Exploratory factor analysis results

As shown in Table 4, this study utilized SPSS 24.0 for exploratory factor analysis, and the results of Kaiser-Meyer-Olkin (KMO) test, Bartlett’s sphere test of the data were examined, which finally showed that the KMO values of the variables were higher than the threshold value of 0.5, and the significance of Bartlett’s sphere test were less than 0.05, indicating that the results of Bartlett’s sphere test for all variables were significant, which could be a reasonable basis for factor analysis of the data (Kaiser, 1974). Therefore, the variables were further factor analyzed using principal component analysis. The results showed that each variable could only extract a factor with an eigenroot greater than 1. The cumulative variance explained was higher than 50%, indicating that the factors analyzed in this study could explain each variable better. Meanwhile, the commonality of all items is more significant than 0.5, and the factor loading is greater than 0.6. All are within the range of values suggested by previous authors (Kohli et al., 1998). To summarize, this study concluded that the findings are of good uni-conformity.

**Table 4:** Exploratory factor analysis results.

Dimension	Items	KMO	Bartlett Sphere Test	Factor Loading	Commonality	Eigenvalue	Total variation explained%
SA	SA1	0.688	0.000	0.864	0.747	2.072	69.050%
	SA2			0.817	0.667		
	SA3			0.811	0.657		
NV	ID1	0.685	0.000	0.793	0.629	2.033	67.751%
	ID2			0.853	0.727		
	ID3			0.823	0.677		
RL	RL1	0.819	0.000	0.815	0.665	2.848	71.193%
	RL2			0.859	0.738		
	RL3			0.866	0.750		
	RL4			0.834	0.696		
CL	CL1	0.797	0.000	0.820	0.672	2.700	67.493%
	CL2			0.849	0.722		
	CL3			0.793	0.629		
	CL4			0.823	0.677		
IR	IR1	0.684	0.000	0.847	0.718	2.025	67.504%
	IR2			0.831	0.691		
	IR3			0.785	0.616		
RC	RC1	0.692	0.000	0.843	0.711	2.154	71.786%
	RC2			0.814	0.663		
	RC3			0.883	0.780		
RA	RA1	0.723	0.000	0.859	0.737	2.232	74.392%
	RA2			0.862	0.742		
	RA3			0.867	0.752		
TF	TF1	0.833	0.000	0.845	0.713	2.934	73.339%
	TF2			0.866	0.750		
	TF3			0.868	0.753		
	TF4			0.847	0.718		
IM	IM1	0.693	0.000	0.859	0.738	2.162	72.071%
	IM2			0.881	0.776		
	IM3			0.805	0.648		
RP	RP1	0.818	0.000	0.807	0.651	2.723	68.076%
	RP2			0.820	0.672		
	RP3			0.854	0.729		
	RP4			0.819	0.671		
CE	BE1	0.884	0.000	0.850	0.722	3.613	72.269%
	BE2			0.850	0.722		
	BE3			0.838	0.702		
	BE4			0.837	0.701		
	BE5			0.875	0.766		
AE	AE1	0.845	0.000	0.880	0.774	3.007	75.179%
	AE2			0.861	0.741		
	AE3			0.865	0.748		
	AE4			0.863	0.745		
EE	EE1	0.715	0.000	0.849	0.720	2.184	72.783%
	EE2			0.866	0.750		
	EE3			0.844	0.713		
SF	SF1	0.727	0.000	0.868	0.754	2.265	75.512%
	SF2			0.873	0.762		
	SF3			0.866	0.749		
BI	BI1	0.706	0.000	0.824	0.679	2.229	74.291%
	BI2			0.887	0.787		
	BI3			0.873	0.762		

### 3.6 Construct validity and reliability

The current study utilized SmartPLS 4.0 to derive Cronbach’s alpha and composite reliability values to indicate internal consistency between items and constructs. The study showed that the values of Cronbach’s alpha coefficient and composite reliability were greater than 0.7, indicating that the data collection instrument was internally consistent with the items (Hair et al., 2020; Almufarreh and Arshad, 2023), and as shown in Table 5, the values of Cronbach’s alpha coefficient and composite reliability both passed, proving that the items for each variable were internally consistent and reliable. Second, this study measured construct validity by extracting the average variance, and the literature suggests that the AVE values are at least 0.5 or higher, assuming that each variable meets the criterion for construct validity (Hair et al., 2020; Almufarreh and Arshad, 2023), as shown in Table 5, the AVE values are all greater than 0.5, indicating good construct validity.

**Table 5:** Construct validity and reliability.

	Cronbach’s alpha	Composite reliability (rho_a)	Composite reliability (rho_c)	Average variance extracted (AVE)
AE	0.89	0.89	0.924	0.752
CE	0.904	0.905	0.929	0.723
BI	0.826	0.828	0.896	0.743
CL	0.839	0.839	0.892	0.675
EE	0.813	0.815	0.889	0.728
NV	0.762	0.762	0.863	0.677
IM	0.805	0.808	0.885	0.721
IR	0.759	0.761	0.862	0.675
RA	0.828	0.829	0.897	0.744
RC	0.803	0.803	0.884	0.718
RL	0.865	0.867	0.908	0.712
RP	0.843	0.844	0.895	0.681
SA	0.775	0.775	0.87	0.69
SF	0.838	0.838	0.902	0.755
TF	0.879	0.879	0.917	0.733

### 3.7 Results of the test of differential validity

Distinguishing validity refers to the difference between different latent variables, which can be tested by comparing the correlation coefficients between the latent variables with the square root of AVE according to Fornell’s suggestion, if the AVE square root is higher than the correlation coefficients of the variables, this indicates that the scale has an excellent distinguishing validity (Fornell and Larcker, 1981). As shown in Table 6, the AVE square root values for each latent variable are more significant than the correlation coefficients between the variables, so the scale has good discriminant validity between the variables.

**Table 6:** Discriminant validity.

	AE	BI	CE	CF	CL	EE	IM	IR	NV	RA	RC	RL	RP	SA	SF	SQ	TF	TM
AE	0.867																	
BI	0.784	0.862																
CE	0.714	0.613	0.85															
CF	0.931	0.796	0.899	0.78														
CL	0.563	0.595	0.489	0.58	0.821													
EE	0.839	0.814	0.688	0.902	0.537	0.853												
IM	0.693	0.612	0.681	0.754	0.486	0.685	0.849											
IR	0.443	0.459	0.55	0.538	0.476	0.465	0.499	0.822										
NV	0.543	0.488	0.515	0.574	0.475	0.504	0.485	0.503	0.823									
RA	0.723	0.706	0.667	0.76	0.528	0.688	0.697	0.586	0.512	0.862								
RC	0.733	0.7	0.607	0.744	0.628	0.707	0.634	0.505	0.483	0.768	0.847							
RL	0.561	0.542	0.563	0.618	0.61	0.566	0.534	0.534	0.573	0.531	0.56	0.844						
RP	0.702	0.652	0.594	0.724	0.498	0.699	0.689	0.551	0.472	0.696	0.651	0.554	0.825					
SA	0.543	0.514	0.592	0.614	0.389	0.533	0.577	0.503	0.526	0.551	0.536	0.439	0.494	0.831				
SF	0.808	0.8	0.665	0.83	0.519	0.822	0.675	0.435	0.5	0.71	0.698	0.52	0.684	0.521	0.869			
SQ	0.826	0.771	0.744	0.865	0.61	0.8	0.866	0.601	0.555	0.889	0.849	0.63	0.86	0.621	0.798	0.745		
TF	0.771	0.71	0.713	0.81	0.55	0.729	0.796	0.504	0.489	0.763	0.719	0.579	0.713	0.575	0.734	0.92	0.856	
TM	0.686	0.673	0.693	0.752	0.788	0.673	0.661	0.755	0.774	0.691	0.703	0.848	0.661	0.703	0.643	0.776	0.696	0.647

### 3.8 VIF test results

Table 7 shows the results of the multicollinearity test for the structural model, showing that the VIF values of all variables are less than 3.3, indicating that there is no multicollinearity problem between the variables.

**Table 7:** VIF test results.

	VIF
SF -> BI	1
TM -> CL	1
TM -> NV	1
TM -> IR	1
TM -> RL	1
TM -> SA	1
TM -> CF	2.514
SQ -> IM	1
SQ -> RA	1
SQ -> RC	1
SQ -> RP	1
SQ -> TF	1
SQ -> TM	1
SQ -> CF	2.514
CF -> AE	1
CF -> CE	1
CF -> EE	1
CF -> SF	1

### 3.9 Q^2^ test results

Q^2^ was used to measure the predictive relevance of the exogenous variables to the endogenous variables, as shown in Table 8, the Q^2^ ranges are all between 0.336–0.645, greater than the critical value of 0, indicating that the model has a strong predictive relevance.

**Table 8:** Q^2^ test results.

	SSO	SSE	Q^2^ (=1-SSE/SSO)
AE	1,700	604.198	0.645
CE	2,125	898.478	0.577
BI	1,275	675.548	0.47
CL	1,700	997.938	0.413
EE	1,275	529.268	0.585
NV	1,275	763.771	0.401
IM	1,275	592.414	0.535
IR	1,275	791.702	0.379
RA	1,275	532.497	0.582
RC	1,275	622.603	0.512
RL	1,700	838.589	0.507
RP	1,700	853.854	0.498
SA	1,275	846.904	0.336
SF	1,275	619.571	0.514
TF	1,700	654.28	0.615

### 3.10 Results of structural model analysis

In the analysis of the structural model, R-square aims to understand the contribution of the variance of the independent variables to the variance of the dependent variable; according to previous studies, R-square values greater than 0.33 are better explanations, as shown in the table, the R-square values are all greater than 0.33, in addition, the larger the value of R-square adjusted, the better the explanatory ability of exogenous variables to endogenous variables; Secondly, Q^2^ is used to measure the predictive relevance of exogenous variables to endogenous variables, and the table presents that the range of Q^2^ are all between 0.336–0.645, which is greater than the critical value of 0, which indicates that the predictive relevance of the model is more potent, i.e., the structural model has a better fit to the research data. Again, the GOF value of goodness-of-fit reflects the model’s ability to estimate the path relationship between variables, with 0.100, 0.250, and 0.360 indicating that the model’s estimation ability is low, medium, and high, respectively (Hair et al., 2012). As shown in Table 9, the GOF value of goodness-of-fit is 0.567, greater than 0.360, indicating the model’s estimation ability is high.

**Table 9:** Results of structural model analysis.

	R-square	R-square adjusted	Q^2^ (=1-SSE/SSO)	GOF
AE	0.866	0.866	0.572	0.567
CE	0.808	0.808	0.577	
BI	0.64	0.64	0.468	
CL	0.62	0.62	0.451	
EE	0.813	0.813	0.439	
NV	0.6	0.599	0.35	
IM	0.75	0.749	0.428	
IR	0.569	0.568	0.346	
RA	0.79	0.79	0.467	
RC	0.721	0.72	0.424	
RL	0.719	0.718	0.51	
RP	0.74	0.74	0.46	
SA	0.494	0.493	0.374	
SF	0.688	0.688	0.488	
TF	0.847	0.847	0.545	
TM			0.354	
SQ	0.602	0.601	0.499	
CF	0.764	0.763	0.544	

### 3.11 Results of hypothesis testing

**Figure 2:**
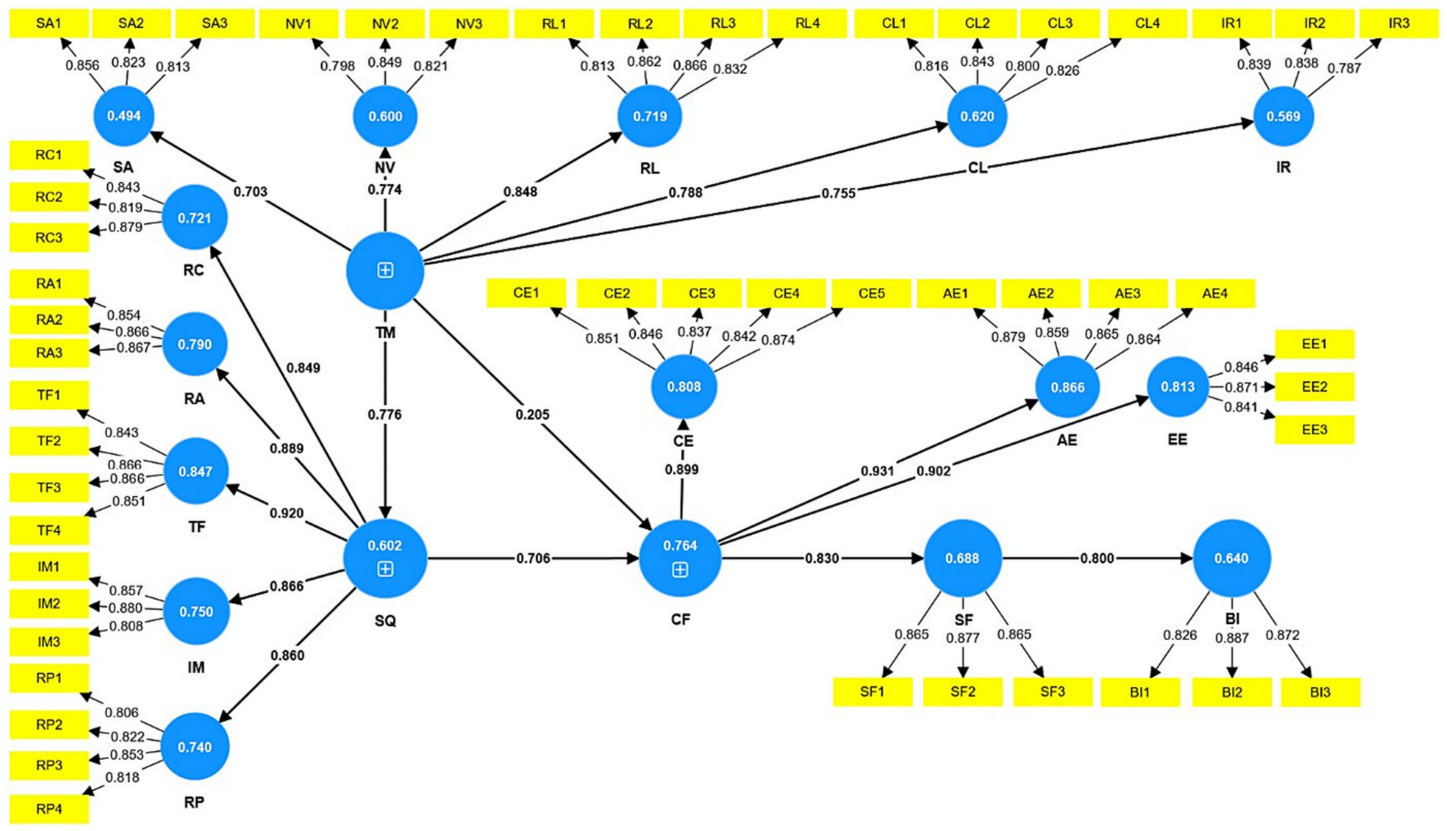
Measurement model.

In this study, PLS-SEM was used to perform hypothesis testing and calculate the path coefficients. Figure 2 is measurement model. In addition, bootstrap method was utilized to self-sample 5,000 times, as the results in Table 10 show that all the paths in this study have *p*-values less than 0.05, which indicates that all the hypotheses are statistically significant, and therefore all the hypotheses in this study have been validated.

**Table 10:** Hypothesis test results.

Hypothesis	Path	Co-efficient	T statistics	*p* values	Decision
H1	TM -> SQ	0.776	32.107	0	Accepted
H2	SQ -> CF	0.706	14.436	0	Accepted
H3	TM-> CF	0.205	4.044	0	Accepted
H4	CF -> SF	0.83	39.555	0	Accepted
H5	SF -> BI	0.8	31.04	0	Accepted
	TM -> CL	0.788	33.889	0	Accepted
	TM -> NV	0.774	28.835	0	Accepted
	TM -> IR	0.755	23.536	0	Accepted
	TM -> RL	0.848	55.258	0	Accepted
	TM -> SA	0.703	22.401	0	Accepted
	SQ -> IM	0.866	61.843	0	Accepted
	SQ -> RA	0.889	66.984	0	Accepted
	SQ -> RC	0.849	45.15	0	Accepted
	SQ -> RP	0.86	30.259	0	Accepted
	SQ -> TF	0.92	98.151	0	Accepted
	CF -> AE	0.931	121.191	0	Accepted
	CF -> CE	0.899	67.513	0	Accepted
	CF -> EE	0.902	73.942	0	Accepted

## 4 Discussion

The validation of the structural equation modeling and the results of the various tests provided some key findings, which are discussed below:

H1 and H2 hold, representing that tourism motivation positively affects service quality (H1), and service quality positively affects the level of expectation confirmation (H2). Among them, H1 has been confirmed by several studies (Lee et al., 2011; Hsieh et al., 2015; Thawornwiriyatrakul and Meeprom, 2020). Most tourists are attracted by the rich and unique resource conditions (RC), such as the historical and cultural background of the heritage parks, fresh and exciting recreational activities (RA), and well-constructed tourist facilities (TF) when choosing a tourist destination (Kim and Eves, 2012; Maaiah and Wouhoush, 2020). The destination’s management (IM) and related personnel (RP), on the other hand, are concerned with the reasonableness of charges, the convenience, and socialization of the tour, and are factors that travelers will focus on prior to their trip (Dwyer et al., 2000). Tourists have psychological expectations of the above services or facilities before they travel, and the degree of their expectations will naturally affect their subsequent experience and feeling of the real service quality. Therefore, understanding the motivation of tourists is also an important factor for the tourism sector to measure how to improve and build the quality of service, and the impact of tourism motivation on service quality is very critical.

Several research findings confirm that H2 holds true, i.e., high quality of service will lead to a pleasurable tourism experience (Hsieh et al., 2015). Abundant resources will bring aesthetic (AE) and spiritual satisfaction to tourists, adequate facilities will make their travel activities (CE) more convenient and comfortable, and the positive communication and friendly interaction of staff in the recreational activities (RA) organized by theme parks will increase the fun of the overall experience of tourists (Dong and Siu, 2013). At the same time, when having a quality amusement experience, the tourists will share positive travel feelings with their family or friends, which is a crucial factor influencing their emotional experience (EE) during the trip.

H3 holds for the extent to which tourism motivation (TM) positively influences expectation confirmation (tourism experience) (CF). Firstly, tourism motivation shapes tourists’ expectations, attitudes and behaviors. Tourism motivation (TM) is the initial expectation of tourists about the sense of the experience they want to acquire; whether it is relaxation (RL), cultural exploration (CE), or interactive socialization (IR), these motivations influence tourists’ perceptions and evaluations of the overall tourism experience (Suhartanto et al., 2020). Secondly, the initial motivation will influence the choice of destination, such as natural or humanistic landscapes. The differences between locations will also influence how tourists perceive the experience. If the travel experience is aligned with the reason why they traveled in the first place, they will be more inclined to participate and enjoy the experience, which will increase their positive moods (Dong and Siu, 2013).

H4 was established to represent that tourists’ sense of travel experience can directly affect their evaluation of travel satisfaction (Jeong and Shin, 2020). Firstly, tourists will experience different living environments, local customs, and lifestyles during their travels, which can enrich their spiritual life; secondly, themed buildings and landscape environments in the parks will provide tourists with a unique aesthetic experience (AE), and authentic experiences in the environments and rich cultural backgrounds will also increase the value of the trip and make the travel experience more meaningful (McIntosh and Prentice, 1999); in addition, positive travel experiences can provide travelers with emotional value and evoke positive emotions such as pleasure and relaxation. Therefore, if travelers’ overall sense of travel experience is of quality, it will positively impact their satisfaction (SF) (Rajaratnam et al., 2014).

H5 holds true, which suggests that satisfaction (SF) has a positive effect on tourists’ post-trip behavioral intentions (BI). Tourists who are satisfied with their travel experience are the ones who are more likely to consider revisiting the same destination in the future, and positive experiences are more likely to elicit thoughts of wanting to revisit or explore the destination again (Tian-Cole and Cromption, 2003). Additionally, travelers who are satisfied with their trip are more likely to become advocates of the destination. Because of the positive experiences they have had, they are more likely to share their relatively positive travel experiences with family and friends around them and have the intention of recommending them to others (Huang and Hsu, 2009).

The analysis of the comprehensive results shows that the path coefficients of tourism motivation (TM)- service quality (SQ), service quality (SQ)-expectation confirmation (CF) degree-satisfaction (SF)-post-trip behavioral intention (BI) are the highest, which shows that tourism motivation, tourism service quality and experience are very important for tourists’ satisfaction and post-trip behavioral intention. In addition, the path coefficient of the tourism motivation-expectation confirmation degree is the lowest, which may be due to the complexity of the tourism experience, tourism experience may be affected by uncertainties such as service quality, travel companions, and unexpected events, which leads to part of the tourism motivation to a certain extent to be covered by deeper aspects of the feelings. On the other hand, differences in destination attributes, age of tourists, and cultural backgrounds can also lead to different perceptions of tourists, thus affecting the actual tourism experience and creating a gap with initial expectations.

Overall, however, the hypotheses are valid, suggesting that tourism motivation is the initial idea and driving force for people to undertake a tourism activity, and secondly is the perception of the overall service quality, both lead to the perception of the tourism experience. And then the overall satisfaction evaluation through the tourism experience, and concluding with the impact of satisfaction on post-trip behavioral intentions, which ultimately triggers future behaviors (Cole and Scott, 2004).

## 5 Theoretical contributions

This study tested the hypothesized relationships between the constructs and validated the factors influencing tourists’ satisfaction and willingness to revisit based on the model path. The findings proved that perceived quality is higher than tourists’ expectations and is an important factor in enhancing experience perception and satisfaction as well as ensuring the willingness to revisit. This suggests that service quality in tourism is an essential element in the study of tourists’ experience, and it is theoretically valuable to analyze it further and explore its component structures. Secondly, tourists’ expectations of the tourism process have a significant impact on the experience, and numerous studies have demonstrated that satisfaction consists of the gap between tourists’ expectations and their actual feelings. From a theoretical point of view, this study once again shows the importance of meeting tourists’ expectations of tourism in terms of service quality (Hui et al., 2007).

On the other hand, the modeling framework of this study runs through the whole process of travel activities before and after the end of the trip, from the expectations before the trip, to the perception of the service quality during the trip, and the resulting experience feelings, and finally the results of satisfaction and intention to revisit, which are composed of the above factors. This provides a more complete theoretical framework for the future tourism industry to analyze a series of tourism influencing factors generated by tourists’ expectations. At the same time, this study proposes a series of valuable structures for this theoretical model based on tourism experience research, providing more possible research directions for the tourism industry.

## 6 Management contributions

Based on the results of quantitative analysis, this paper puts forward some suggestions for the actual management of historical and cultural theme parks. First, historical and cultural theme parks should strengthen their attractiveness. For example, it focuses on discovering and publicizing the theme park’s unique historical and cultural background, combining short video advertisements, narrating historical stories and other publicity methods. Conveying historical and cultural information to visitors more interestingly may create positive perceptions and expectations of the theme park, thus encouraging them to visit. The next step is to improve the service quality of the park, which directly affects tourists’ expectations and experiences. Positive expectations are the key to determining whether tourists will generate tourism behavior, and experiencing service quality is essential for tourists to confirm their expectations. High expectations can also lead to too large a difference between expectations and reality, thus reducing the sense of experience and satisfaction (Bigné et al., 2005). Therefore, the quality of service is very critical through strengthening the professional training of staff, enriching the interpretation system in the park to enhance the understanding and emotional connection of visitors to the theme park content, regularly surveying and collecting feedback, and evaluation of visitors to the theme park, and analyzing and improving the suggestions made by visitors can help the park to enhance the quality of service. In addition, enriching the performance programs in the park, with different performances corresponding to different folk cultures or historical stories, and developing more entertainment experience programs according to the various ages and cultural backgrounds of tourists can also enrich the content of the park and attract tourists with different attributes, to bring positive expectations to the tourists and improve the quality of the park’s services. On the other hand, the sense of experience is an important factor in determining satisfaction, and satisfaction is also the key to ensuring a positive willingness to revisit. Therefore, the authenticity and the cleanliness of the park should be ensured. The integrity and beauty of the theme buildings and landscaping in the park are easy to evoke emotional and aesthetic responses in tourists, which can help to enhance the sense of immersion and satisfaction of tourists, and tourists can improve the overall sense of tourism experience and satisfaction when their emotional and aesthetic demands are met. Whether or not desired outcomes are realized is an important factor in determining whether or not a strategy is successful and encourages tourists to make a second choice (Ahrholdt et al., 2019). The purpose of this study is to provide recommendations for the construction and development of historical and cultural theme parks. Based on the perspective of practical management, this study establishes reasonable and effective reference suggestions for the development of historical and cultural theme parks.

## 7 Conclusion, research limitations, and future research directions

### 7.1 Conclusion

Starting from the perspective of Expectation Confirmation Theory and based on the specific characteristics of historical and cultural theme parks, and based on expectation (tourism motivation), perceived performance (service quality), degree of expectation confirmation (tourism experience), satisfaction and post-trip behavioral intention, this study establishes a brand new research model, explores the specific influencing factors between the model structures, and through the research and analysis of the intrinsic relationship between each of these constructs, it reflects the article’s understanding of the changes in the perceptions of the tourists before and after the experience, and at the same time proves that each of these constructs has a direct or indirect influence on the satisfaction and post-trip behavioral intention of the tourists. Therefore, relevant practitioners should satisfy tourists’ expectations and needs from the three perspectives of understanding the motivation of tourism, strengthening service quality, and emphasizing the tourists’ experience, so as to enhance satisfaction and the possibility of revisiting.

### 7.2 Research limitations and future research directions

Undeniably, this study also has some limitations, and at the same time, these limitations represent possible directions for future research:

1. The historical and cultural theme park selected in the article is located in a city with deep history and culture, but the current economic development is relatively backward, if it is in a city with better economic development, the findings may be different. In the future, more developed cities can be selected for case studies or comparative studies.
2. Although there is a positive effect of service quality on travel motivation, the significance is not vital in comparison with the results of the other data, and there may be moderating variables between the two that affect the relationship between travel motivation and travel experience, for example, individual differences, cultural backgrounds, or previous travel experiences may moderate the way in which travel motivation is transformed into an actual travel experience, and the reasons for this are worthy of more in-depth research.
3. The investigation scope of this study only selected a historical and cultural heritage park as the research location, which is small in scope, therefore, the investigation scope can be expanded in the future research, to broaden the research horizons and make the study results more persuasive.

## Data availability statement

The original contributions presented in the study are included in the article/supplementary material, further inquiries can be directed to the corresponding author.

## Ethics statement

Ethical review and approval was not required for the study on human participants in accordance with the local legislation and institutional requirements. Written informed consent from the patients/ participants or patients/participants legal guardian/next of kin was not required to participate in this study in accordance with the national legislation and the institutional requirements.

## Author contributions

LY: Investigation, Software, Writing – original draft, Writing – review & editing. AM: Supervision, Writing – review & editing.
